# Glycosomal ABC transporter 3 (*GAT3*) deletion enhances the oxidative stress responses and reduces the infectivity of *Trypanosoma cruzi*

**DOI:** 10.1371/journal.pntd.0013479

**Published:** 2025-09-11

**Authors:** Davi Alvarenga Lima, Héllida Marina Costa-Silva, Karen Stephanie Sebe Albergaria, Juliana Martins Ribeiro, Daniela de Melo Resende, Bruno Alves Santarossa, Daniel Barbosa Liarte, Simone Guedes Calderano, Silvane Maria Fonseca Murta

**Affiliations:** 1 Grupo Genômica Funcional de Parasitos, Instituto René Rachou, Fiocruz Minas, Belo Horizonte, Minas Gerais, Brazil; 2 Laboratório de Ciclo Celular, Instituto Butantan, São Paulo, Brazil; 3 Departamento de Biologia, Universidade Federal do Piauí, Teresina, Brazil; McGill University, CANADA

## Abstract

Glycosomes, peroxisome-like organelles in *Trypanosoma cruzi*, contain enzymes involved in various metabolic processes, including glycolysis. Glycosomal ABC transporters (GATs) play a vital role in maintaining metabolic homeostasis by facilitating metabolite exchange between glycosomes and the cytoplasm. GAT3 is a member of the GAT family, which also includes *GAT1* and *GAT2*. *GAT3* transcript levels are downregulated in benznidazole-resistant *T. cruzi* populations; however, its specific functions remain unknown. Therefore, in this study, we generated *GAT3* single-knockout and null mutant lines of the *T. cruzi* Dm28c strain using the CRISPR/Cas9 system to investigate GAT3 roles in parasite biology. RT-qPCR revealed increased *GAT2* transcript levels in the *GAT3* null mutant line, without any changes in *GAT1* levels. Our findings suggest that *GAT3* is not essential for *T. cruzi* survival, as null mutant parasites showed no growth difference compared to the Cas9-expressing controls. Moreover, the *GAT3* single-knockout line exhibited increased resistance to benznidazole, whereas the null mutant line exhibited benznidazole susceptibility similar to the control. Furthermore, both *GAT3* single-knockout and null mutant lines showed increased tolerance to hydrogen peroxide-induced oxidative stress. *In vitro* infection assay of L929 murine fibroblasts revealed that the *GAT3* null parasites exhibited a significantly lower infection rate and fewer intracellular amastigotes than the controls. Overall, *GAT3* is crucial for *T. cruzi* infectivity and the regulation of oxidative stress responses, playing key roles in the metabolic regulation and pathogenicity of this parasite.

## Author summary

In this study, we investigated the roles of glycosomal ABC transporter 3 (GAT3) in *Trypanosoma cruzi*, the causative agent of Chagas disease. GAT3 is a member of a transporter family crucial for metabolic process regulation in glycosomes. Here, we used the CRISPR/Cas9 system to generate *GAT3* single-knockout and null mutant lines of *T. cruzi* Dm28c to understand the GAT3 functions. We found that *GAT3* was not essential for parasite survival, as both GAT3 mutant *T. cruzi* lines exhibited normal growth patterns similar to the parental line. However, the mutants exhibited enhanced tolerance to hydrogen peroxide-induced oxidative stress. Moreover, the absence of GAT3 impaired the ability of the parasite to infect the host cells, as evidenced by the fewer infected fibroblasts and intracellular amastigotes. These results underscore the importance of GAT3 for the regulation of oxidative stress responses and parasite infectivity and highlight its potential as a therapeutic target for Chagas disease.

## Introduction

Chagas disease, caused by the flagellated protist *Trypanosoma cruzi*, is a significant public health issue in Latin America, with an estimated 6 million people infected and over 70 million living in endemic areas [1]. The geographical spread of this disease has increased owing to climate change and migration patterns, altering its vector habitats and increasing human exposure [2,3]. Transmission primarily occurs via faeces of the infected triatomine insects (*Reduviidae* family); however, other transmission modes, including congenital transmission, blood transfusion, organ transplantation, and contaminated food ingestion, have also gained attention in recent years [4].

Currently, *T. cruzi* infection is treated with two drugs, benznidazole (BZ) and nifurtimox, which exhibit limited efficacy, particularly during the chronic phase of the disease. These drugs are also associated with adverse side effects, often leading to treatment discontinuation [5]. Variations in the genetic diversity of *T. cruzi* strains and their hosts partially explain the different efficacies of existing trypanocidal drugs [5]. Furthermore, high genomic plasticity of *T. cruzi* poses significant challenges for its treatment, resulting in frequent therapeutic failures [6,7].

Recent transcriptomic analyses comparing BZ-susceptible (17WTS) and BZ-resistant (17LER) *T. cruzi* populations have revealed a set of genes involved in the metabolic processes associated with BZ resistance [8]. Among the differentially expressed genes, we focused on glycosomal ABC transporter 3 (*GAT3*), as its functions in *T. cruzi* remain ambiguous.

Glycosomes, peroxisome-derived organelles, are essential for various metabolic pathways in trypanosomatids. These organelles contain enzymes involved in glycolysis and other metabolic processes. Protein import into glycosomes is mediated by peroxisomal targeting signals (*PTS1* and *PTS2*), which direct soluble enzymes to the organelle matrix, and by membrane peroxisomal targeting signals (mPTS), which are recognized by cytosolic factors such as PEX19 to mediate targeting of membrane proteins, including those of the glycosomal membrane [9–11]. Metabolite transport across the glycosomal membrane is facilitated by glycosomal transporters, which ensure the distribution of essential substrates, such as glucose-6-phosphate, NAD, and dihydroxyacetone phosphate, in the glycosomes and cytosol, thereby supporting cellular homeostasis [12,13]. Biochemical studies and proteomic analyses have shown that *T. cruzi* glycosomes are multifunctional organelles involved in both catabolic (e.g., glycolysis) and anabolic processes (e.g., fatty acid synthesis) [14,15].

Three half-size Glycosomal ABC Transporters (*GAT1*, *GAT2*, and *GAT3*) have been identified in *T. brucei* [13]. These transporters belong to subgroup D of the ABC transporter family and are characterised by a single transmembrane domain containing six predicted transmembrane helices, along with a nucleotide-binding domain responsible for ATP binding and hydrolysis, which provides the energy for transport [16,17]. RNA interference-mediated depletion of GAT1 and GAT3 in procyclic *T. brucei* cells does not impair their cell growth in a glucose-containing medium [18]. In contrast, depletion of GAT1, but not GAT3, is lethal under glucose-free conditions. Therefore, GAT1 acts as a fatty acid transporter, specifically mediating the uptake of oleoyl-CoA into *T. brucei* glycosomes. However, the specific functions of GAT3 in *T. brucei* remain unknown [18]. Homologous sequences corresponding to **T. brucei* GATs* have also been identified in *T. cruzi* [17]. However, their specific functional roles in *T. cruzi* remain largely unknown.

Glycosomal enzymes play key roles in energy production, oxidative stress response, and reactive oxygen species (ROS) detoxification, showing potential as therapeutic targets [15]. BZ-resistant *T. cruzi* populations exhibit enhanced antioxidant defence mechanisms, including the overexpression of tryparedoxin peroxidase and ascorbate peroxidase, which protect these parasites from oxidative stress [19–21].

In this study, we investigated the roles of *GAT3* in *T. cruzi* via clustered regularly interspaced palindromic repeat (CRISPR)/CRISPR-associated protein 9 (Cas9)-mediated gene knockout (KO). We also assessed the impacts of *GAT3* disruption on the parasite growth rate, BZ and hydrogen peroxide (H₂O₂) susceptibilities, and infectivity using L929 fibroblasts. Through these analyses, we aimed to elucidate the functional roles of *GAT3* in parasitic metabolism, oxidative stress tolerance, and parasite infectivity.

## Methods

### Parasite cultivation

Epimastigote forms of the *T. cruzi* Dm28c strain were cultured at 27 °C in the liver infusion tryptose (LIT) medium supplemented with 10% heat-inactivated foetal bovine serum (Gibco, Thermo Fisher Scientific, Waltham, MA, USA) [20]. The culture was maintained through weekly passages by inoculating 2 × 10⁶ parasites into 5 mL of the medium. All experiments were conducted using epimastigotes in the logarithmic growth phase.

### Genomic and functional domain analyses of the GAT family in the *T. cruzi* DM28c strain

Amino acid sequences of the glycosomal transporter family (TcGATs) of the *T. cruzi* Dm28c strain were retrieved from TriTrypDB (https://tritrypdb.org/tritrypdb/app) and aligned using the MAFFT algorithm v.7.49 with *maxiterate* and *localpair* arguments to improve the alignment accuracy [22,23]. The resulting multiple sequence alignments were analysed to assess the sequence identity and similarity. Alignment statistics, including sequence length, alignment length, number of gaps, gap length, identity, similarity, and percentage change, were calculated using the *infoalign* tool in the EMBOSS package v.6.6 [24].

Amino acid sequences were subjected to *InterProScan* 5.72 analysis with default parameters to identify the conserved functional domains among TcGAT proteins. The identified domains were mapped onto the multiple sequence alignments using TcGAT1 as the reference sequence [25].

Multiple sequence alignments were visualised using the UniPro Ugene Toolkit, with domain annotations mapped onto the aligned sequences. Conserved and variable residues were highlighted based on the sequence conservation scores (S1 Fig). The final alignment figure was generated to illustrate the localisation of the functional domains and degree of sequence conservation among TcGAT1 (C4B63_44g213), TcGAT2 (C4B63_2g366), and TcGAT3 (C4B63_19g205) [26].

### Generation of GAT3 *T. cruzi* KO lines

We used the CRISPR/Cas9 system to generate GAT3 *T. cruzi* null mutants, as previously described [27]. Plasmid pLEW13 containing geneticin as a resistance marker was used to express SpCas9 and T7RNAP. Epimastigote forms of the *T. cruzi* Dm28c strain carrying this plasmid successfully expressing Cas9, gently provided by Dr. Simone Calderano (Cell Cycle Laboratory, Instituto Butantan, Brazil), were transfected with the donor DNAs and guide RNA templates selected using the Eukaryotic Pathogen CRISPR guide RNA/DNA Design Tool [28] based on the **T. cruzi* GAT3* gene sequence annotated as C4B63_19g205 in TriTrypDB [23,29]. Guide RNAs were transcribed *in vivo* from a double-stranded DNA template produced via polymerase chain reaction (PCR), where the forward primer included the T7 RNA polymerase promoter, target cleavage sequence, and region complementary to the 3′-end of the reverse primer. Primers used in this study are listed in S1 Table.

### Reverse transcription-quantitative PCR (RT-qPCR)

Epimastigote forms of *T. cruzi* (approximately 10⁸ cells) were harvested and resuspended in 1 mL of TRIzol Reagent (Invitrogen, Thermo Fisher Scientific, Waltham, MA, USA). Total RNA was extracted using chloroform. After DNase I (Ambion, Thermo Fisher Scientific, Austin, TX, USA) treatment, cDNA was synthesised using the Superscript II reverse transcriptase (Invitrogen), following the manufacturer’s instructions. All cDNA samples were diluted to 30 ng/μL and used for RT-qPCR amplification reactions with the 1 × SYBR GREEN master mix (Applied Biosystems, Thermo Fisher Scientific, Foster City, CA, USA) and specific primers (S1 Table). Housekeeping gene DNA polymerase I was used for normalisation. Amplification was performed using the QuantStudio 12 KFlex system (Thermo Fisher Scientific, Waltham, MA, USA) under the following PCR conditions: 95 °C for 10 min, followed by 40 cycles of denaturation at 95 °C for 15 s and annealing/extension at 60 °C for 1 min. Fluorescence levels were measured after each extension step. Fold-change was calculated using the comparative CT (2^−ΔΔCT^) method [30].

### Growth curve of epimastigote forms

To evaluate parasite growth, epimastigote forms of *T. cruzi* (1 × 10⁶ parasites/mL) were inoculated into the LIT medium, and parasite counts were determined daily over ten days using the Z1 Coulter Particle Counter (Beckman Coulter, Brea, CA, USA).

### BZ and H₂O₂ susceptibility assays

To evaluate the BZ and H₂O₂ susceptibilities of control parasites expressing Cas9 and GAT3 single-KO and null *T. cruzi* mutant lines, susceptibility assays were performed as described below. Briefly, 2 × 10⁶ epimastigote forms were incubated at 27 ºC with 1 mL LIT medium containing different concentrations of BZ (3.8–30.7 μM) and H₂O₂ (300–700 μM) in a 24-well plate. After seven days, parasite counts in the absence and presence of each drug were determined using the Z1 Coulter Particle Counter (Beckman Coulter). Then, 50% growth inhibitory concentration (EC_50_) was calculated using the non-linear regression variable slope model with the GraphPad Prism v.9.5.0 software.

### L929 fibroblast infection

Trypomastigotes were obtained by infecting the L929 mouse fibroblasts with aged parasite cultures (15 days after reaching the stationary phase). The cells were placed at a humidified incubator with 5% CO_2_ at 37 °C, after 24-h incubation, fibroblasts were washed with phosphate-buffered saline (PBS) to remove any non-infective parasites, and RPMI medium supplemented with 0.2% sodium bicarbonate and 20% non-inactivated horse serum (Gibco, Thermo Fisher Scientific, Waltham, MA, USA) was added to eliminate the epimastigote forms. After 24 h, the medium containing horse serum was replaced with the RPMI medium supplemented with foetal bovine serum and 10% non-inactivated horse serum. After ten days, the bottles were transferred to an incubator with 5% CO_2_ at 33 °C to promote the release of trypomastigote forms into the supernatants. For the *in vitro* infection experiment, L929 fibroblasts were counted with a Neubauer chamber and plated in a 24-well plate with glass coverslips (20,000 cells/well). After 24 h, these fibroblasts were infected with the trypomastigotes recovered from the supernatant at a ratio of 10 parasites/fibroblasts for 2 h. Parasites that failed to infect the cells were removed by washing with PBS, and the infected fibroblasts were then incubated in RPMI-1640 medium. Parasitic infectivity was evaluated after 48 h. The slides were stained with Rapid Panoptic (Laborclin, Pinhais, PR, Brazil) and photographed. Infection was assessed by counting the number of infected fibroblasts and intracellular amastigotes using the ImageJ software (National Institutes of Health, Bethesda, MD, USA).

### Identification of GATs-interacting drugs and assessment of their trypanocidal activities

DrugBank (https://go.drugbank.com/) is a widely used database providing detailed information on drugs, including small molecules, their action mechanisms, therapeutic targets, pharmacokinetic properties, and adverse effects [23]. This database is an essential resource for drug repositioning research, offering structured data on drug–target interactions, biochemical pathways, and therapeutic indications.

The GAT1, GAT2 and GAT3 protein sequences were obtained and compared with the molecular targets available on DrugBank using the Basic Local Alignment Search Tool (BLAST) [31]. Proteins with the highest similarity to GATs were selected, and their associated drugs were identified. The drug list was filtered using the following criteria: Approval for human use, described action mechanism, and clinical conditions. Following screening, three compounds were selected for EC_50_ assays: α-Tocopherol (Sigma-Aldrich, Merck, St. Louis, MO, USA), glimepiride (European Pharmacopoeia, EDQM, Strasbourg, France), and bumetanide (Sigma). Our in silico analysis indicated that all three selected compounds were predicted to interact not only with GAT3 but also with GAT1 and GAT2, albeit with differing levels of sequence identity and binding site coverage. These findings suggest that the compounds may target conserved functional domains shared among the GAT family members.

*In vitro* anti-*T. cruzi* activities of the three drugs against amastigote and trypomastigote forms were evaluated using L929 mouse fibroblasts infected with the Tulahuen strain of the parasite expressing the *Escherichia coli* β-galactosidase as a reporter gene, as described previously [32]. Results are expressed as the percentage of *T. cruzi* growth inhibition. BZ at its EC_50_ (1 µg mL^-1 ^= 3.8 µM) was used as a positive control. Moreover, alamarBlue dye (Invitrogen) was used to determine the cytotoxic concentration reducing the L929 cell viability by 50% (CC_50_) [32]. EC_50_ and CC_50_ values were determined via linear interpolation, and the selectivity index was calculated as the ratio of CC_50_ L929 cells/EC_50_ *T. cruzi.*

### Statistical analyses

Data were analysed using the GraphPad Prism v.9.5.0 software. Statistical comparisons between Cas9 and mutant parasites were performed via ordinary one-way or two-way analysis of variance (ANOVA), followed by Bonferroni’s post-hoc test. Statistical significance was set at *p* < 0.05. *p*-values are expressed using the GraphPad Prism conventions as follows: *ns* (*p* > 0.05), *p < 0.05, **p < 0.01, ***p < 0.001, and ****p < 0.0001.

## Results

### Genomic and functional domain analyses of the GAT family in the *T. cruzi* DM28c strain

Multiple sequence alignment revealed distinct degrees of identity and similarity among GAT1, GAT2, and GAT3. GAT3 of the *T. cruzi* DM28c strain, exhibited 32.8% sequence identity with GAT1 and 28% sequence identity with GAT2, with similarity values of 49.2% and 47.6%, respectively. Meanwhile, GAT1 exhibited 27.1% identity and 47.5% similarity with GAT2 (S1 Fig). These differences suggest that, despite sharing a common evolutionary origin [17], GAT3 may have undergone functional diversification during the evolution of *T. cruzi*.

*InterProScan* analysis identified the following three conserved domains shared by all three proteins: ATP-binding cassette subfamily D (IPR050835), ABC transporter type 1 transmembrane domain (IPR011527), and ABC transporter-like ATP-binding domain (IPR003439; S1 Fig).

### Confirmation of GAT3 allele deletion in *T. cruzi* mutant lines

*T. cruzi* Dm28c strain genome contains a single copy of *GAT3* (TriTrypDB accession number C4B63_19g205) located on chromosome 39 in the CL Brener Esmeraldo-like strain (not assigned to a chromosome in the Dm28c strain). This gene spans 1,995 bp and encodes a protein comprising 664 amino acids. To generate *GAT3* single-KO and null mutant lines, we used the CRISPR/Cas9 system as previously described [27].

To generate a single-KO line (*TcGAT3*^*+/-*^), a donor DNA cassette derived from the pJET BLAST plasmid was inserted into the genome of *TcCas9* parasites. This cassette contained sequences homologous to the 5′- and 3′-untranslated regions (UTRs) of *GAT3*, along with blasticidin S deaminase (BSD) for selection. Integration into the parasite genome occurred via homologous recombination following sgRNA-directed cleavage at the target site, replacing one *GAT3* allele. Correct integration of the BSD cassette into the *T. cruzi* genome was confirmed via PCR analysis of the TcGAT3^+/-^ *T. cruzi* mutant line (S2A and S2B Fig). Additional PCR was performed using *GAT3*-specific primers. The results indicated the presence of *GAT3* in the mutant TcGAT3^+/-^ parasites, confirming that only one allele was replaced (S2C Fig).

**Fig 1:**
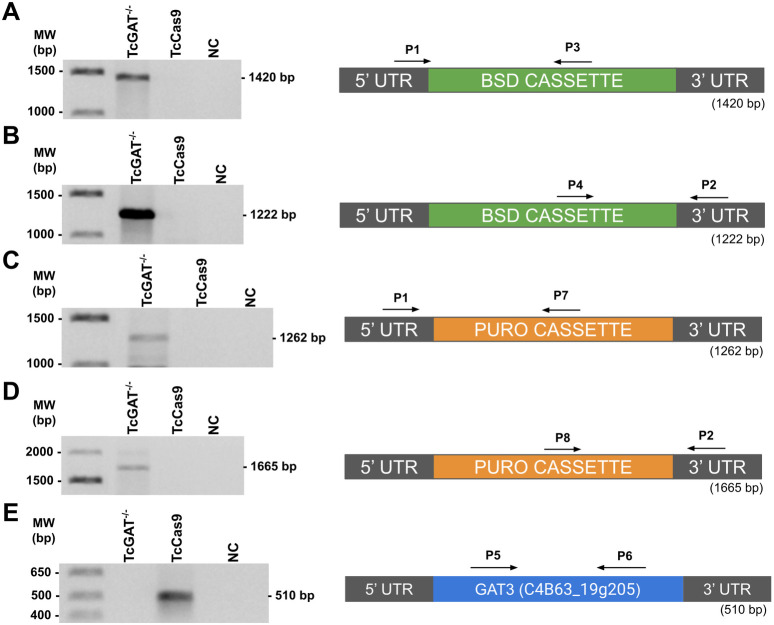
Characterization of *Trypanosoma cruzi* glycosomal ABC transporter 3 (GAT3)-knockout (KO) lines generated using the CRISPR/Cas9 system. Correct integration of the resistance markers, (A and B) blasticidin S deaminase (BSD; 1420 bp and 1222 bp) and (C and D) puromycin (PURO; 1262 bp and 1665 bp), was evaluated via PCR by annealing a primer in the 3′- or 5′- UTR adjacent to the cassette (primer P1 or P2) and another within each resistance marker sequence (primers P3 or P4 for BSD and P7 or P8 for PURO). (E) Fragment *GAT3*-coding sequence (510 bp) was amplified via PCR using the P5 and P6 primers. MW, molecular weight; NC, negative control; bp, base pair.

To generate *GAT3* KO cell lines, a donor DNA cassette derived from the ptPURO plasmid was inserted into the genome of TcGAT3^+/-^ parasites. This cassette contained sequences homologous to the 5′- and 3′-UTRs of *GAT3*, along with the puromycin (PURO) resistance gene for selection. Correct integration of both the BSD and PURO cassettes into the *T. cruzi* genome was confirmed via PCR analysis of the mutant TcGAT3^-/-^ *T. cruzi* line (Fig 1A–1D). PCR was also used to verify *GAT3* deletion in these parasites. No amplification was observed, confirming the successful replacement of both *GAT3* alleles in the mutant TcGAT3^-/-^ line (Fig 1E).

### *GAT3* deletion increases the GAT2 transcript levels

**Fig 2:**
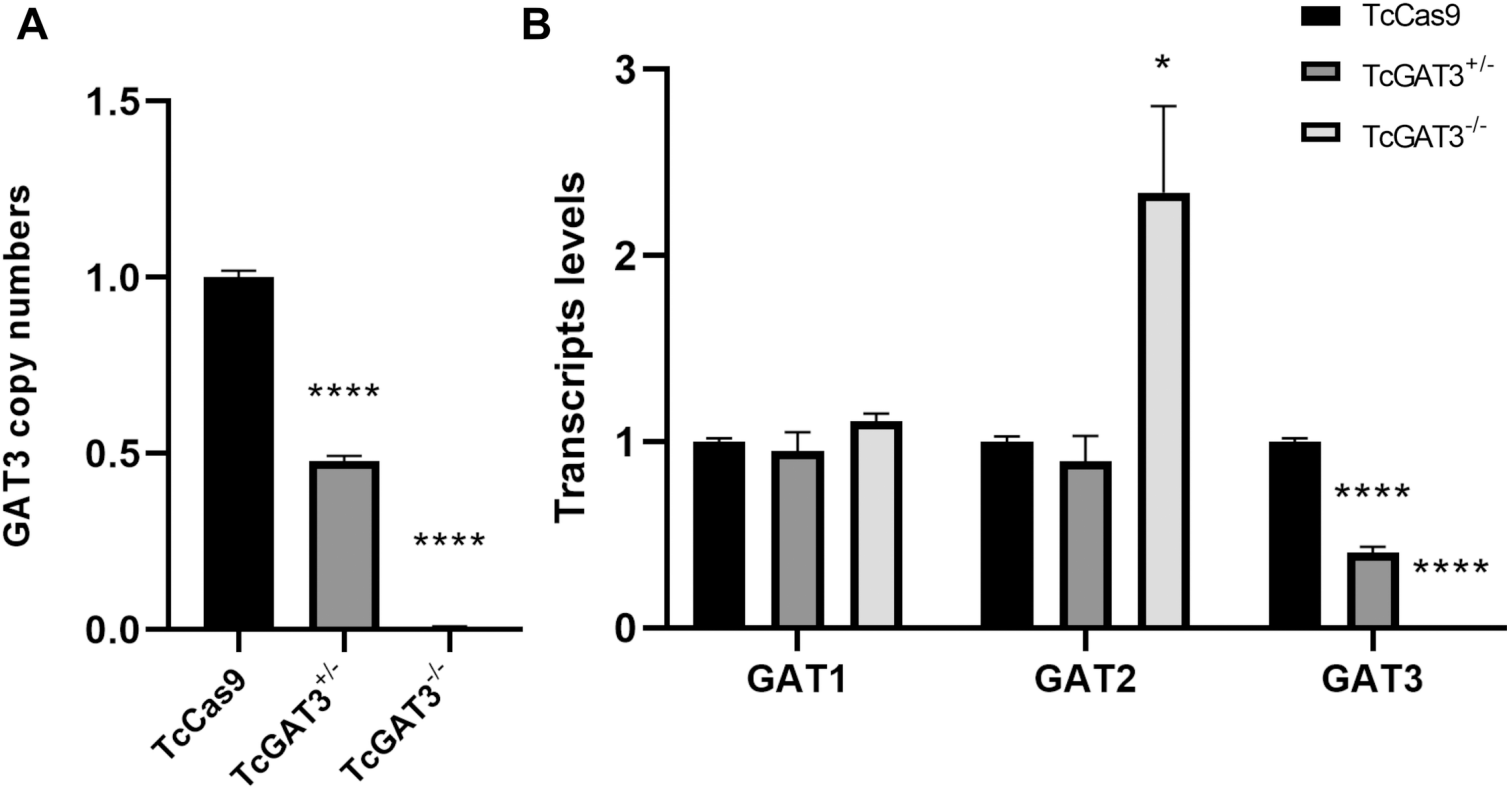
Evaluation of *GAT* copy numbers and transcript levels in the wild-type and *GAT3*-mutant epimastigote forms of *T. cruzi.* qPCR was performed using specific primers to determine the *GAT3* gene copy numbers and expression levels. Copy number was determined using the 2 ⁻ ^ΔΔCT^ method and normalized to that of the constitutive gene, DNA polymerase I. (A) *GAT3* copy numbers in the control (TcCas9), single-KO (TcGAT3 ⁺ /⁻), and KO (TcGAT3 ⁻ /⁻) parasites. (B) Transcript levels of *GAT1*, *GAT2*, and *GAT3* in the same parasite lines. Statistical analysis was conducted via one-way ANOVA, followed by Dunnett’s post-hoc test, to compare the control (TcCas9) and mutant (TcGAT3 ⁺ /⁻ and TcGAT3 ⁻ /⁻) lines. Asterisks indicate the statistically significant differences relative to the control (*p* < 0.05; ****p < 0.0001).

Consistent with the PCR results, gene copy number analysis confirmed the complete deletion of *GAT3* in the null mutant *T. cruzi* line (Fig 2A). *GAT3* transcripts were undetectable in the same line (Fig 2B). Moreover, a 50% reduction in both the *GAT3* copy number and expression level was observed in the single-KO mutant line (Fig 2A and 2B).

*GAT3* is a member of the *GAT* family, which also includes *GAT1* and *GAT2*. To investigate whether *GAT3* deletion in *T. cruzi* affects other family members, transcript levels of *GAT1* (C4B63_44g213) and *GAT2* (C4B63_2g366) were assessed via qPCR using cDNAs of the control (TcCas9) and mutant *T. cruzi* lines. RT-qPCR analysis revealed that *GAT2* transcript levels in the *GAT3*-KO line were 2.2-fold higher than those in the Cas9-expressing parasites (Fig 2B). Notably, *GAT1* transcript levels remained unchanged in all *T. cruzi* parasite lines (Fig 2B).

### *GAT3* deletion does not affect the parasite growth

Growth of the epimastigote forms of Cas9-expressing and *GAT3* single-KO and null mutant *T. cruzi* lines was assessed by counting the parasites every 24 h. No significant difference in growth was observed between the Cas9-expressing and *GAT3* mutant *T. cruzi* lines. These results suggest that GAT3 is not essential for *T. cruzi* survival, as its deletion did not affect the parasite’s growth *in vitro* (S3 Fig).

### *GAT3* deletion alters the BZ and H₂O₂ susceptibilities of the parasites

**Fig 3:**
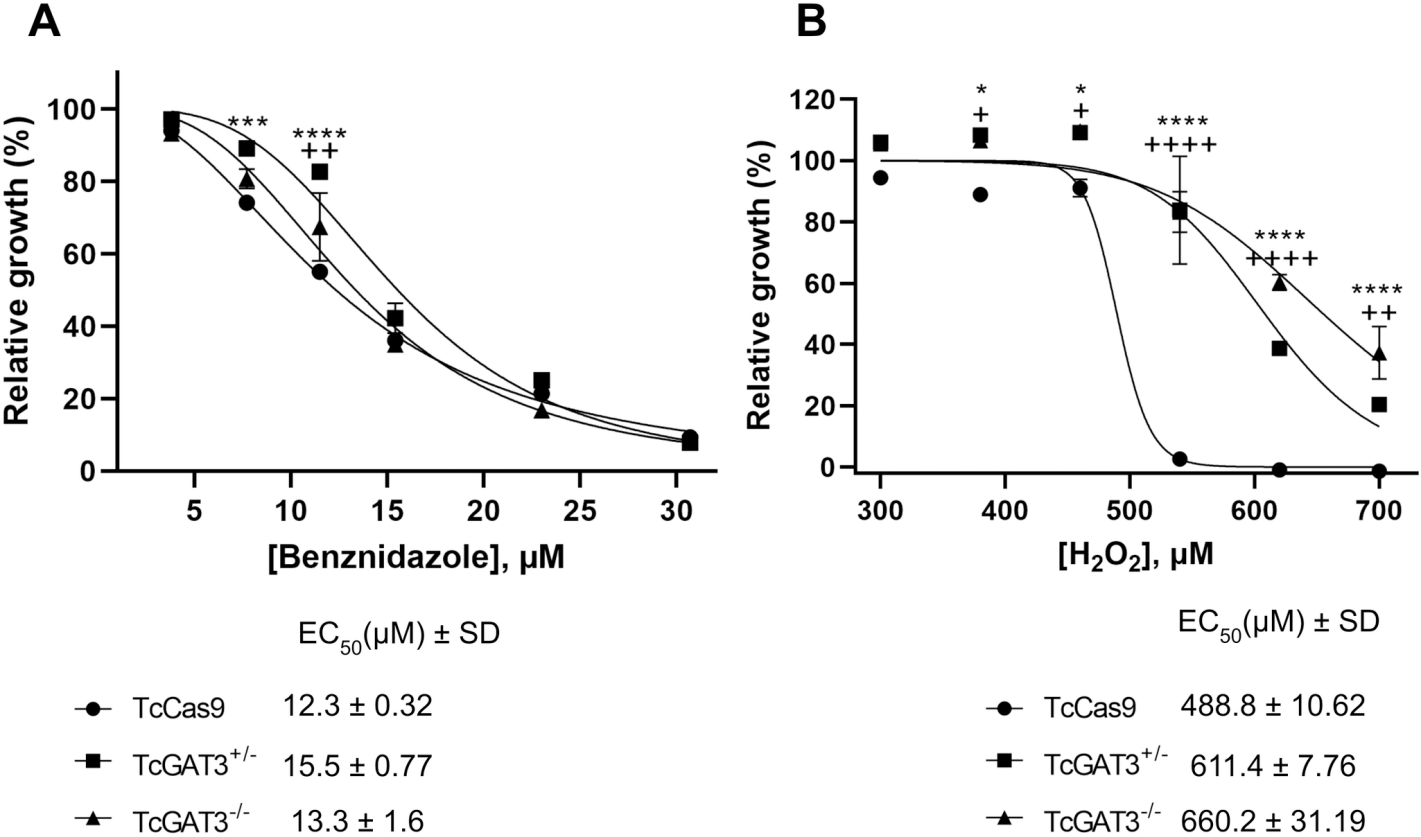
*In vitro* susceptibility assessment of the epimastigote forms of the Cas9-expressing control (*TcCas9)*, *GAT3 hemi-*KO, and *GAT3-*KO *T. cruzi* lines. Parasites were cultured in the presence of different concentrations of **(A)** benznidazole (3.8–30.7 μM) and **(B)** hydrogen peroxide (300–700 μM). Their growth was determined after seven days of incubation with or without the drugs. Data represent the means with standard deviations of three independent experiments performed in triplicate. The 50% growth inhibitory concentration (EC_50_) was determined using the non-linear regression-variable slope model with the GraphPad Prism v.9.5.0 software. A two-way ANOVA test, followed by the Bonferroni post-hoc test, was used to compare the TcCas9 parasites and mutants for each drug concentration. Differences between the Cas9-expressing controls and single-KO *TcGAT3*^*+/-*^ line: *p < 0.05, **p < 0.01, ***p < 0.001, and ****p ≤ 0.0001; differences between the Cas9-expressing controls and GAT3-null *TcGAT3*^*-/-*^ line: ^+^*p <* 0.05, ^++^*p <* 0.01, ^+++^*p* < 0.001, and ^++++^**p* *≤ 0.0001.

We investigated whether *GAT3* deletion affects the BZ and H₂O₂ susceptibilities of the mutant parasites. *GAT3* single-KO TcGAT3^+/-^ mutant line exhibited a 1.26-fold higher resistance to BZ than the control line (TcCas9; Fig 3A). However, the *GAT3*-null mutant *T. cruzi* line exhibited an EC₅₀ value similar to that of the control parasites. Specifically, for BZ, EC₅₀ values were 12.3 and 13.3 µM for the control and GAT3-KO TcGAT3^-/-^ *T. cruzi* lines, respectively, whereas the *GAT3* single-KO TcGAT3^+/-^ mutant line exhibited an EC₅₀ value of 15.5 µM (Fig 3A).

We also assessed H₂O₂ susceptibility and found that the deletion of one *GAT3* allele or complete loss of *GAT3* increased the parasite tolerance to oxidative stress. The EC₅₀ value of the control (TcCas9) parasites was 488.8 µM, whereas those of the TcGAT3^+/-^ and TcGAT3^-/-^ mutant *T. cruzi* lines were 611.4 and 660.2 µM, respectively. Therefore, TcGAT3^+/-^ and TcGAT3^-/-^ mutant *T. cruzi* lines showed 1.25- and 1.35-fold higher EC₅₀ values than those of the TcCas9 controls, respectively (Fig 3B).

### Absence of *GAT*3 reduces the percentage of infected fibroblasts and the number of intracellular amastigotes

**Fig 4:**
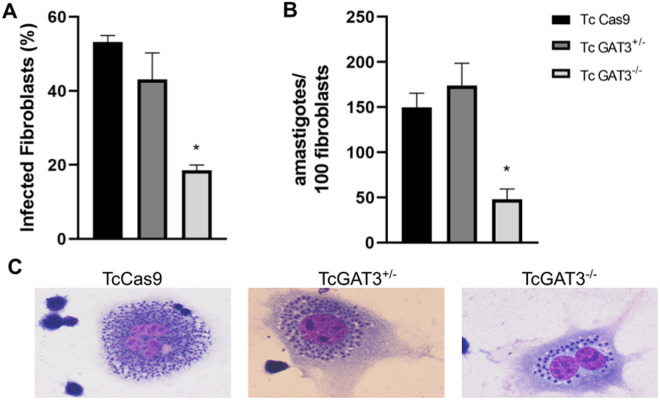
Infectivities of the control and GAT3-mutant *T. cruzi* parasites. L929 fibroblasts were infected with the trypomastigote forms of the control parasites expressing Cas9 and *TcGAT3*^*+/-*^ and *TcGAT3*^*-/-*^ *T. cruzi* mutant lines at a ratio of 1:10 and evaluated 48 h post-infection. The graph shows the number of intracellular amastigotes per 100 fibroblasts 48 h after infection. (A) Percentage of infected L929 cells. (B) Number of intracellular amastigotes per 100 fibroblasts. Data represent the means with standard deviation of two independent experiments performed in triplicate. Statistical analyses were conducted via ordinary one-way ANOVA, followed by Tukey’s post-hoc test, to compare the TcCas9, TcGAT3^+/-^, and TcGAT3^-/-^ *T. cruzi* lines. *p < 0.05 vs. Cas9-expressing control. (C) Representative images of the infected Tc*Cas9*, Tc*GAT3*^*+/-*^, and Tc*GAT3*^*-/-*^ cells stained with Rapid Panoptic (Laborclin). Images were captured using the Zeiss fluorescence microscope with a 63 × objective lens.

To assess the impact of *GAT3* deletion on *T. cruzi* infectivity, L929 mouse fibroblasts were infected with the *TcCas9* control parasites and *TcGAT3*^*+/-*^ and *TcGAT3*^*-/-*^ *T. cruzi* mutant lines. Deletion of one *GAT3* allele did not affect the percentage of infected fibroblasts and number of intracellular amastigotes compared to those in the TcCas9 parasites (Fig 4A and 4B). However, both the percentage of infected fibroblasts and the number of intracellular amastigotes were significantly decreased in the *GAT3*-null (TcGAT3^⁻/⁻^) *T. cruzi* line compared to those in the Cas9-expressing control parasites 48 h post-infection (Fig 4A and 4B). GAT3-null mutant parasites exhibited 18.6% infected fibroblasts and an average of 47.5 amastigotes per 100 fibroblasts, whereas the *TcCas9* controls exhibited 53% infected fibroblasts and 149.5 amastigotes per 100 fibroblasts.

### *In vitro* trypanocidal activities of *GATs*-interacting drugs

Potential GATs-interacting drugs were tested against both the amastigote and trypomastigote forms of the *T. cruzi* Tulahuen strain expressing the *E. coli* β-galactosidase as a reporter gene, as described previously [32]. Bumetanide and α-tocopherol were inactive and showed no detectable trypanocidal effects at the tested concentrations (20–400 μM; S2 Table). Glimepiride (5.0–203 μM) exhibited an EC₅₀ value of 15.6 μM, which was 4-fold higher than that of the reference drug, BZ (EC₅₀ = 3.8 μM; S2 Table). However, it was cytotoxic to the L929 mouse fibroblasts, with a CC₅₀ value of 96.3 μM. This drug has not yet been approved for *in vivo* testing in mouse models owing to its low selectivity for parasites, with a selectivity index of 6.16.

## Discussion

Glycosomes are specialised peroxisome-like organelles found in *T. cruzi* and other kinetoplastids. They contain enzymes involved in various metabolic pathways, including glycolysis, pentose phosphate, β-oxidation of fatty acid, and purine salvage pathways [15]. GATs play a crucial role in maintaining metabolic homeostasis by regulating the exchange of metabolites between glycosomes and cytoplasm. The GAT family comprises GAT1, GAT2, and GAT3. In *T. brucei*, depletion of GAT1, but not GAT3, was lethal to procyclic cells cultured in a glucose-free medium [18]. GAT1 depletion effects revealed its function as a fatty acid transporter with a selective affinity for oleoyl-CoA; however, specific roles of GAT3 in *T. brucei* remain unknown [18]. Homologous sequences corresponding to *T. brucei* GATs have been reported in *T. cruzi* [17]. However, their specific functional roles in these parasites remain unknown.

Among the differentially expressed transcripts in BZ-resistant *T. cruzi* populations, the *GAT3* levels were downregulated [8]. In this study, we provide the first evidence of the impacts of *GAT3* deletion on *T. cruzi* phenotypes using the CRISPR/Cas9 technology.

We successfully generated *GAT3* single-KO and null mutant lines of *T. cruzi* Dm28c using the CRISPR/Cas9 system. Our data indicated that *GAT3* was not essential for *T. cruzi* survival under *in vitro* conditions, as *GAT3*-null parasites exhibited no alterations in growth rate compared to the Cas9-expressing controls (S3 Fig). These results are consistent with those of a previous study, in which GAT3 depletion via RNA interference did not affect the *in vitro* growth of *T. brucei* [18].

Multiple amino acid sequence alignment revealed distinct degrees of identity and similarity among GAT1, GAT2, and GAT3 in the *T. cruzi* DM28c strain. The results showed low sequence identity (approximately 30%) and similarity (approximately 47%) despite the presence of conserved domains in all three proteins: ATP-binding cassette sub-family D (IPR050835), ABC transporter type 1 transmembrane domain (IPR011527), and ABC transporter-like ATP-binding domain (IPR003439; S1 Fig). These data, together with the phylogenetic analysis of glycosomal proteins from kinetoplastid and diplonemid protists performed by Andrade-Alviárez et al. (2022), suggest an early evolutionary divergence, possibly driven by functional specialization. The observed low conservation rates may reflect differences in substrate transport across the glycosomal membrane [17].

In our study, the *GAT3*-null mutant *T. cruzi* line completely lacked *GAT3* gene copies and transcripts. No alterations in *GAT1* transcript levels were observed in this line. However, *GAT2*, another glycosomal transporter, exhibited increased transcript levels, possibly as a compensatory response to *GAT3* loss. This suggests that *GAT2* and *GAT3* perform overlapping or similar roles in substrate transport. However, since gene expression in trypanosomatids is primarily regulated at the post-transcriptional level, it remains to be determined whether the increased GAT2 transcript levels also lead to elevated protein expression or enhanced transport activity. Future studies addressing these aspects will be important to clarify the extent of functional compensation between these transporters. In *T. brucei*, RNA interference-mediated depletion of *GAT1* increased the GAT3 protein levels, indicating *GAT3* upregulation as a compensatory response to mitigate the adverse impacts of *GAT1* loss [18].

Currently, the action mechanism of BZ remains unclear. Metabolomic studies suggest that its trypanocidal effect is mediated by the production of low-molecular-weight thiols toxic to parasites [33]. Other proposed mechanisms involve the generation of reactive species, such as glyoxal, which, if not neutralised, compromises the survival of *T. cruzi* [34,35]. Moreover, BZ promotes the oxidation of nucleotides in the *T. cruzi* nucleotide pool, and their incorporation by DNA polymerases leads to mutagenesis [36].

In our study, deletion of one *GAT3* allele in *T. cruzi* (*TcGAT3*^*+/-*^) resulted in a 50% reduction in *GAT3* transcript levels, increasing the parasites resistance to BZ. However, complete deletion of *GAT3* (*TcGAT3*^*-/-*^) did not alter BZ susceptibility, as these parasites exhibited an EC_50_ value similar to that of the control (*TcCas9*) parasites. We hypothesised that the compensatory upregulation of *GAT2* plays a crucial role in restoring the BZ susceptibility of *GAT3*-null mutant *T. cruzi* parasites. These findings are consistent with the transcriptome analysis results, indicating that BZ-resistant *T. cruzi* parasites exhibit downregulated *GAT3* transcript levels [8]. Increased BZ resistance of the *GAT3* single-KO line suggests that the downregulation of this transporter plays a role in BZ resistance. We propose that the TcGAT3 ⁻ / ⁻ *T. cruzi* line activates compensatory mechanisms, including the upregulation of GAT2, to restore glycosomal function and metabolic homeostasis disrupted by the complete loss of GAT3. This compensation likely restores the ability of the parasite to efficiently transport BZ-derived metabolites or maintain redox and metabolic balance, thereby preserving BZ susceptibility. In contrast, in the TcGAT3 ⁺ / ⁻ line, GAT3 levels are reduced but not absent, and there is no activation of GAT2 expression. This intermediate state may impair the efficient transport of key metabolites without triggering full compensatory mechanisms, leading to an altered intracellular environment that diminishes the efficacy of BZ-derived trypanocidal metabolites—thus increasing resistance. Similar resistance mechanisms have been reported for the other genes in *T. cruzi*. For instance, reduced hexose transporter activity suggests that altered transporter function remodels the cellular metabolism and stress–response pathways in drug-resistant *T. cruzi* [37]. Deletion of *NTR1*, a nitroreductase involved in drug activation, confers increased resistance to nitroaromatic compounds [38]. These findings highlight the complex drug resistance mechanisms and remarkable adaptability of *T. cruzi*.

The ABC transporter family, to which GAT3 belongs, plays a crucial role in the environmental stress responses of trypanosomatids. These proteins directly contribute to stress adaptation by acting as efflux pumps and eliminating metabolites or indirectly by maintaining the intracellular homeostasis [39]. Previous studies revealed that BZ-resistant trypanosomatids exhibit duplication of the *ABCG1* gene, an ABC transporter of the G subfamily [40].

We previously demonstrated that *T. cruzi* populations with *in vitro*-induced resistance to BZ are protected against oxidative stress through a mechanism involving the overexpression of tryparedoxin peroxidase, ascorbate peroxidase, and other antioxidant defence enzymes, including iron superoxide dismutase [19–21]. We investigated the susceptibility of the *GAT*3 mutant *T. cruzi* parasites to H_2_O_2_, which induces oxidative stress. Both *GAT3* single-KO and null mutant *T. cruzi* lines were more tolerant to H_2_O_2_ than the *Tc*Cas9 controls. Glycosomes contain key enzymes detoxifying ROS and protecting *T. cruzi* from oxidative damage [41]. Thus, the partial or complete loss of GAT3 may enhance oxidative stress tolerance by reducing the transport of toxic metabolites into the glycosome or altering the flux of essential metabolic intermediates. These changes could trigger metabolic adaptations that promote antioxidant responses or limit the activity of ROS-generating pathways. Among the main antioxidant enzymes present in glycosomes are iron-dependent superoxide dismutases (FeSOD-B1 and -B2), which neutralize superoxide radicals, and non-selenium glutathione peroxidases, which provide resistance to hydroperoxides [42].

In this study, *GAT3* deletion in *T. cruzi* reduced the percentage of infected fibroblasts and the number of intracellular amastigotes compared to those in the control parasites 48 h post-infection. GAT3 is likely involved in host cell infection, possibly through its role in glycosomal function. As glycosomes contain key enzymes for energy metabolism and reactive oxygen species (ROS) detoxification, the efficient transport of substrates into this organelle is essential for maintaining metabolic and redox balance during infection [15,43]. We hypothesize that GAT3 may be more effective than GAT2 at transporting specific substrates necessary for processes directly linked to host cell invasion and intracellular survival. While the upregulation of GAT2 observed in GAT3-deficient parasites may provide partial metabolic compensation, it appears insufficient to fully restore the transport efficiency required for optimal infection. Consequently, the absence of GAT3 likely impairs key metabolic pathways, reducing the parasite’s ability to establish and maintain infection despite preserved viability.

Energy metabolism is crucial for the intracellular proliferation of *T. cruzi*, and *GAT3* deletion significantly impacts metabolite transport to glycosomes [44]. Recently, glycosomal enzymes and their functions have garnered increasing attention because of their potential as therapeutic targets and the limited knowledge regarding their roles [14,43]. Additionally, the GAT family has emerged as a promising therapeutic target; however, the specific metabolites transported by these proteins, particularly by GAT2 and GAT3, remain largely unknown. Our study revealed the relationship between *GAT3* levels and parasite susceptibility to BZ and H₂O₂, as well as the impacts of *GAT3* deletion on parasite infectivity and intracellular proliferation. Previous studies have suggested glycosome-associated proteins as promising therapeutic targets and revealed the downregulated *GAT3* levels in BZ-resistant *T. cruzi* populations [8,45].

In this study, we identified three drugs (bumetanide, α-tocopherol, and glimepiride) potentially interacting with all three *T. cruzi* GAT family members by searching DrugBank. *In vitro* activities of these drugs against both the trypomastigote and amastigote forms of *T. cruzi* were evaluated. Bumetanide and α-tocopherol were ineffective against *T. cruzi*. In contrast, glimepiride exerted trypanocidal effects (EC_50_ = 15.6 µM); however, its selectivity for this parasite was low. The tested drugs did not yield promising results. However, as glycosomal transporters are essential for parasite survival and differ from mammalian transporters, they remain promising drug targets for the selective disruption of *T. cruzi* metabolism.

This study revealed the significant roles of *GAT3* in *T. cruzi* oxidative stress tolerance, drug susceptibility, infectivity, and growth. Our findings suggest that *GAT3* plays critical roles in parasite adaptation to adverse conditions and host infection. However, the precise molecular mechanisms underlying these effects remain unclear. Future studies, including transcriptomic, proteomic, and in-depth functional studies, should determine the specific roles of *GAT3* and assess its potential as a therapeutic target for Chagas disease.

To the best of our knowledge, this study represents the first functional characterisation of *GAT3* in *T. cruzi*, highlighting its roles in parasite metabolism, BZ susceptibility, and infectivity. *GAT3* expression (*TcGAT3*^*+/-*^) increased BZ resistance; however, its complete deletion (*TcGAT3*^*-/-*^) did not affect BZ susceptibility, possibly due to compensatory *GAT2* level upregulation. Moreover, *GAT3*-null mutant parasites exhibited enhanced oxidative stress tolerance and reduced infectivity, further supporting the roles of *GAT3* in intracellular parasite survival and redox homeostasis.

Although our *in vitro* drug screen did not identify any promising inhibitors of *GATs*, glycosomal transporters remain attractive therapeutic targets because of their important roles in parasitic metabolism. Further investigations, including metabolomics and structural studies, are necessary to elucidate the specific substrates transported by *GAT3* and its interactions with other glycosomal pathways. Elucidation of these mechanisms is crucial to develop innovative therapeutic strategies against *T. cruzi* and improve the treatment options for Chagas disease.

## Supporting information

S1 Data (A) EC_50_ for Hydrogen Peroxide. Raw data used to generate the EC₅₀ values shown in Fig 3B. Column A indicates the H₂O₂ concentrations used in the assay, while columns B to J contain the relative growth data from each replicate for the three *T. cruzi* populations. (B) EC₅₀ for Benznidazole. Raw data used to generate the EC₅₀ values shown in Fig 3A. Column A indicates the benznidazole concentrations used in the assay, while columns B to J contain the relative growth data from each replicate for the three *T. cruzi* populations. (C) Infected fibroblasts. Raw data used to generate the infectivity values shown in Fig 4A. Column A indicates the percentage of infected L929 cells, while columns B to G contain the percentage of infected fibroblasts recorded in each assay, performed in two replicates for the three *T. cruzi* populations. (D) Amastigotes in 100 cells. Raw data used to generate the infectivity values shown in Fig 4B. Column A indicates the number of intracellular amastigotes in infected L929 cells, while columns B to G contain the mean number of amastigotes per 100 L929 cells recorded in each assay, performed in two replicates for the three *T. cruzi* populations. (E) RT-qPCR. Raw data used to generate the transcript level values shown in Fig 1B. Column A lists the genes analyzed (GAT1, GAT2, and GAT3), while columns B to J contain the relative quantification data for each replicate from the three *T. cruzi* populations. (F) Genomic qPCR. Raw data used to generate the gene copy number values shown in Fig 1A. Column A lists the genes analyzed (GAT1, GAT2, and GAT3), while columns B to J contain the relative genomic quantification data from each replicate of the three *T. cruzi* populations. (G) Growth curve. Raw data used to generate the growth curve shown in S3 Fig. Column A indicates the 10 days of the experiment, while columns B to J contain the mean number of epimastigote forms counted in each assay, performed in three replicates for the three *T. cruzi* populations.
(XLSX)

S1 Table List of primers used in this study. (DOCX)

S2 Table *In vitro* trypanocidal activity, cytotoxicity, and selectivity index of selected compounds that interact with GATs against Tulahuen *T. cruzi* strain. (DOCX)

S1 Fig Multiple amino acid sequence alignment of TcGAT1, TcGAT2, and TcGAT3 from *T. cruzi* DM28c strain and domain annotation based on TcGAT1. The amino acid sequences of *TcGAT1* (C4B63_44g213), *TcGAT2* (C4B63_2g366), and *TcGAT3* (C4B63_19g205) from *T. cruzi* DM28c strain were aligned using MAFFT, highlighting conserved and variable residues. Conserved residues are shaded in dark gray, while less conserved positions are shaded in lighter gray. The domains were mapped with TcGAT1 as reference onto the alignment: ATP-binding cassette sub-family D domain (purple), ABC transporter type 1 transmembrane domain (red) and ABC transporter-like ATP-binding domain (blue). Gaps introduced during the alignment process are represented by dashes (-). The alignment illustrates the overall sequence conservation among the three transporters and highlights specific variations that may contribute to functional divergence.
(TIF)

S2 Fig Characterization of *T. cruzi GAT3*-single-knockout lines generated using CRISPR/Cas9. Correct integration of the resistance markers, (A and B) blasticidin S deaminase (BSD; 1420 bp and 1222 bp) was evaluated via PCR by annealing a primer in the 3′UTR and 5′UTR region adjacent to the cassette (primer P1 or P2) and another within each resistance marker sequence (primers P3 or P4 for BSD). (C) The fragment *GAT3-*coding sequence (510 bp) was amplified via PCR with the P5 and P6 primers. MW: molecular weight; NC: negative control; bp: base pair.
(TIF)

S3 Fig Growth curve of epimastigote forms of Cas9-expressing control parasites and *GAT3* single-knockout and null mutant *T. cruzi* lines. An initial inoculum of 2 x 10^6^ parasites per mL was prepared for the Cas9 parasites and mutant lines, which were counted every 24 h using the Z1 Coulter Counter, during 10 days.
(TIF)

## References

[1] WHO. Chagas disease. 2025 [cited 22 May 2025]. https://www.who.int/news-room/fact-sheets/detail/chagas-disease-(american-trypanosomiasis)

[2] Reyes C, González CR, Alvarado S, Flores L, Martin C, Oyarce A, et al. Chagas disease in northern Chile: Detection of *Trypanosoma cruzi* in children, dogs and triatomine bugs. Acta Trop. 2022;235:106631. doi: 10.1016/j.actatropica.2022.106631 35948082

[3] Tidman R, Abela-Ridder B, de Castañeda RR. The impact of climate change on neglected tropical diseases: a systematic review. Trans R Soc Trop Med Hyg. 2021;115(2):147–68. doi: 10.1093/trstmh/traa192 33508094 PMC7842100

[4] López-García A, Gilabert JA. Oral transmission of Chagas disease from a One Health approach: A systematic review. Trop Med Int Health. 2023;28(9):689–98. doi: 10.1111/tmi.13915 37488635

[5] Sales Junior PA, Molina I, Fonseca Murta SM, Sánchez-Montalvá A, Salvador F, Corrêa-Oliveira R, et al. Experimental and Clinical Treatment of Chagas Disease: A Review. Am J Trop Med Hyg. 2017;97(5):1289–303. doi: 10.4269/ajtmh.16-0761 29016289 PMC5817734

[6] Reis-Cunha JL, Valdivia HO, Bartholomeu DC. Gene and Chromosomal Copy Number Variations as an Adaptive Mechanism Towards a Parasitic Lifestyle in Trypanosomatids. Curr Genomics. 2018;19(2):87–97. doi: 10.2174/1389202918666170911161311 29491737 PMC5814966

[7] Cruz-Saavedra L, Schwabl P, Vallejo GA, Carranza JC, Muñoz M, Patino LH, et al. Genome plasticity driven by aneuploidy and loss of heterozygosity in *Trypanosoma cruzi*. Microb Genom. 2022;8(6):mgen000843. doi: 10.1099/mgen.0.000843 35748878 PMC9455712

[8] Lima DA, Gonçalves LO, Reis-Cunha JL, Guimarães PAS, Ruiz JC, Liarte DB, et al. Transcriptomic analysis of benznidazole-resistant and susceptible *Trypanosoma cruzi* populations. Parasit Vectors. 2023;16(1):167. doi: 10.1186/s13071-023-05775-4 37217925 PMC10204194

[9] Opperdoes FR. Compartmentation of carbohydrate metabolism in trypanosomes. Annu Rev Microbiol. 1987;41:127–51. doi: 10.1146/annurev.mi.41.100187.001015 3120638

[10] Gatto GJ Jr, Geisbrecht BV, Gould SJ, Berg JM. Peroxisomal targeting signal-1 recognition by the TPR domains of human PEX5. Nat Struct Biol. 2000;7(12):1091–5. doi: 10.1038/81930 11101887

[11] Saveria T, Halbach A, Erdmann R, Volkmer-Engert R, Landgraf C, Rottensteiner H, et al. Conservation of PEX19-binding motifs required for protein targeting to mammalian peroxisomal and trypanosome glycosomal membranes. Eukaryot Cell. 2007;6(8):1439–49. doi: 10.1128/EC.00084-07 17586720 PMC1951143

[12] Guerra DG, Decottignies A, Bakker BM, Michels PAM. The mitochondrial FAD-dependent glycerol-3-phosphate dehydrogenase of Trypanosomatidae and the glycosomal redox balance of insect stages of *Trypanosoma brucei* and Leishmania spp. Mol Biochem Parasitol. 2006;149(2):155–69. doi: 10.1016/j.molbiopara.2006.05.006 16806528

[13] Yernaux C, Fransen M, Brees C, Lorenzen S, Michels PAM. *Trypanosoma brucei* glycosomal ABC transporters: identification and membrane targeting. Mol Membr Biol. 2006;23(2):157–72. doi: 10.1080/09687860500460124 16754359

[14] Acosta H, Burchmore R, Naula C, Gualdrón-López M, Quintero-Troconis E, Cáceres AJ, et al. Proteomic analysis of glycosomes from *Trypanosoma cruzi* epimastigotes. Mol Biochem Parasitol. 2019;229:62–74. doi: 10.1016/j.molbiopara.2019.02.008 30831156 PMC7082770

[15] Quiñones W, Acosta H, Gonçalves CS, Motta MCM, Gualdrón-López M, Michels PAM. Structure, Properties, and Function of Glycosomes in *Trypanosoma cruzi*. Front Cell Infect Microbiol. 2020;10:25. doi: 10.3389/fcimb.2020.00025 32083023 PMC7005584

[16] Theodoulou FL, Holdsworth M, Baker A. Peroxisomal ABC transporters. FEBS Lett. 2006;580(4):1139–55. doi: 10.1016/j.febslet.2005.12.095 16413537

[17] Andrade-Alviárez D, Bonive-Boscan AD, Cáceres AJ, Quiñones W, Gualdrón-López M, Ginger ML, et al. Delineating transitions during the evolution of specialised peroxisomes: Glycosome formation in kinetoplastid and diplonemid protists. Front Cell Dev Biol. 2022;10:979269. doi: 10.3389/fcell.2022.979269 36172271 PMC9512073

[18] Igoillo-Esteve M, Mazet M, Deumer G, Wallemacq P, Michels PAM. Glycosomal ABC transporters of *Trypanosoma brucei*: characterisation of their expression, topology and substrate specificity. Int J Parasitol. 2011;41(3–4):429–38. doi: 10.1016/j.ijpara.2010.11.002 21163262

[19] Nogueira FB, Krieger MA, Nirdé P, Goldenberg S, Romanha AJ, Murta SMF. Increased expression of iron-containing superoxide dismutase-A (TcFeSOD-A) enzyme in *Trypanosoma cruzi* population with in vitro-induced resistance to benznidazole. Acta Trop. 2006;100(1–2):119–32. doi: 10.1016/j.actatropica.2006.10.004 17113553

[20] Nogueira FB, Ruiz JC, Robello C, Romanha AJ, Murta SMF. Molecular characterization of cytosolic and mitochondrial tryparedoxin peroxidase in *Trypanosoma cruzi* populations susceptible and resistant to benznidazole. Parasitol Res. 2009;104(4):835–44. doi: 10.1007/s00436-008-1264-1 19018566

[21] Nogueira FB, Rodrigues JFA, Correa MMS, Ruiz JC, Romanha AJ, Murta SMF. The level of ascorbate peroxidase is enhanced in benznidazole-resistant populations of *Trypanosoma cruzi* and its expression is modulated by stress generated by hydrogen peroxide. Mem Inst Oswaldo Cruz. 2012;107(4):494–502. doi: 10.1590/s0074-02762012000400009 22666860

[22] Rozewicki J, Li S, Amada KM, Standley DM, Katoh K. MAFFT-DASH: integrated protein sequence and structural alignment. Nucleic Acids Res. 2019;47(W1):W5–10. doi: 10.1093/nar/gkz342 31062021 PMC6602451

[23] Alvarez-Jarreta J, Amos B, Aurrecoechea C, Bah S, Barba M, Barreto A, et al. VEuPathDB: the eukaryotic pathogen, vector and host bioinformatics resource center in 2023. Nucleic Acids Res. 2024;52(D1):D808–16. doi: 10.1093/nar/gkad1003 37953350 PMC10767879

[24] Rice PM, Bleasby AJ, Ison JC. EMBOSS user’s guide: practical bioinformatics. Cambridge (G. B.): Cambridge university press. 2011.

[25] Jones P, Binns D, Chang H-Y, Fraser M, Li W, McAnulla C, et al. InterProScan 5: genome-scale protein function classification. Bioinformatics. 2014;30(9):1236–40. doi: 10.1093/bioinformatics/btu031 24451626 PMC3998142

[26] Okonechnikov K, Golosova O, Fursov M, UGENE team. Unipro UGENE: a unified bioinformatics toolkit. Bioinformatics. 2012;28(8):1166–7. doi: 10.1093/bioinformatics/bts091 22368248

[27] Costa FC, Francisco AF, Jayawardhana S, Calderano SG, Lewis MD, Olmo F, et al. Expanding the toolbox for *Trypanosoma cruzi*: A parasite line incorporating a bioluminescence-fluorescence dual reporter and streamlined CRISPR/Cas9 functionality for rapid in vivo localisation and phenotyping. PLoS Negl Trop Dis. 2018;12(4):e0006388. doi: 10.1371/journal.pntd.0006388 29608569 PMC5897030

[28] Peng D, Tarleton R. EuPaGDT: a web tool tailored to design CRISPR guide RNAs for eukaryotic pathogens. Microb Genom. 2015;1(4):e000033. doi: 10.1099/mgen.0.000033 28348817 PMC5320623

[29] Berná L, Rodriguez M, Chiribao ML, Parodi-Talice A, Pita S, Rijo G, et al. Expanding an expanded genome: long-read sequencing of *Trypanosoma cruzi*. Microb Genom. 2018;4(5):e000177. doi: 10.1099/mgen.0.000177 29708484 PMC5994713

[30] Livak KJ, Schmittgen TD. Analysis of relative gene expression data using real-time quantitative PCR and the 2(-Delta Delta C(T)) Method. Methods. 2001;25(4):402–8. doi: 10.1006/meth.2001.1262 11846609

[31] Altschul SF, Gish W, Miller W, Myers EW, Lipman DJ. Basic local alignment search tool. J Mol Biol. 1990;215(3):403–10. doi: 10.1016/S0022-2836(05)80360-2 2231712

[32] Romanha AJ, Castro SL de, Soeiro M de NC, Lannes-Vieira J, Ribeiro I, Talvani A, et al. In vitro and in vivo experimental models for drug screening and development for Chagas disease. Mem Inst Oswaldo Cruz. 2010;105(2):233–8. doi: 10.1590/s0074-02762010000200022 20428688

[33] Trochine A, Creek DJ, Faral-Tello P, Barrett MP, Robello C. Benznidazole biotransformation and multiple targets in *Trypanosoma cruzi* revealed by metabolomics. PLoS Negl Trop Dis. 2014;8(5):e2844. doi: 10.1371/journal.pntd.0002844 24853684 PMC4031082

[34] Docampo R, Moreno SN. Free radical metabolites in the mode of action of chemotherapeutic agents and phagocytic cells on *Trypanosoma cruzi*. Rev Infect Dis. 1984;6(2):223–38. doi: 10.1093/clinids/6.2.223 6328615

[35] Wilkinson SR, Kelly JM. Trypanocidal drugs: mechanisms, resistance and new targets. Expert Rev Mol Med. 2009;11:e31. doi: 10.1017/S1462399409001252 19863838

[36] Rajão MA, Furtado C, Alves CL, Passos-Silva DG, de Moura MB, Schamber-Reis BL, et al. Unveiling benznidazole’s mechanism of action through overexpression of DNA repair proteins in *Trypanosoma cruzi*. Environ Mol Mutagen. 2014;55(4):309–21. doi: 10.1002/em.21839 24347026

[37] dos Santos PF, Ruiz JC, Soares RPP, Moreira DS, Rezende AM, Folador EL, et al. Molecular characterization of the hexose transporter gene in benznidazole resistant and susceptible populations of *Trypanosoma cruzi*. Parasit Vectors. 2012;5:161. doi: 10.1186/1756-3305-5-161 22871258 PMC3431256

[38] Wilkinson SR, Taylor MC, Horn D, Kelly JM, Cheeseman I. A mechanism for cross-resistance to nifurtimox and benznidazole in trypanosomes. Proc Natl Acad Sci U S A. 2008;105(13):5022–7. doi: 10.1073/pnas.0711014105 18367671 PMC2278226

[39] da Costa KM, Salustiano EJ, do Carmo Valente R, Freire-de-Lima L, Mendonça-Previato L, Previato JO. Thiol efflux mediated by an ABCC-like transporter participates for *Trypanosoma cruzi* adaptation to environmental and chemotherapeutic stresses. Cold Spring Harbor Laboratory. 2020. doi: 10.1101/2020.03.26.009753

[40] Petravicius PO, Costa-Martins AG, Silva MN, Reis-Cunha JL, Bartholomeu DC, Teixeira MMG, et al. Mapping benznidazole resistance in trypanosomatids and exploring evolutionary histories of nitroreductases and ABCG transporter protein sequences. Acta Trop. 2019;200:105161. doi: 10.1016/j.actatropica.2019.105161 31494121

[41] Allmann S, Bringaud F. Glycosomes: A comprehensive view of their metabolic roles in *T. brucei*. Int J Biochem Cell Biol. 2017;85:85–90. doi: 10.1016/j.biocel.2017.01.015 28179189

[42] Wilkinson SR, Meyer DJ, Taylor MC, Bromley EV, Miles MA, Kelly JM. The *Trypanosoma cruzi* enzyme TcGPXI is a glycosomal peroxidase and can be linked to trypanothione reduction by glutathione or tryparedoxin. J Biol Chem. 2002;277(19):17062–71. doi: 10.1074/jbc.M111126200 11842085

[43] Durrani H, Hampton M, Rumbley JN, Zimmer SL. A Global Analysis of Enzyme Compartmentalization to Glycosomes. Pathogens. 2020;9(4):281. doi: 10.3390/pathogens9040281 32290588 PMC7237986

[44] Haanstra JR, González-Marcano EB, Gualdrón-López M, Michels PAM. Biogenesis, maintenance and dynamics of glycosomes in trypanosomatid parasites. Biochim Biophys Acta. 2016;1863(5):1038–48. doi: 10.1016/j.bbamcr.2015.09.015 26384872

[45] Barros-Alvarez X, Gualdrón-López M, Acosta H, Cáceres AJ, Graminha MAS, Michels PAM, et al. Glycosomal targets for anti-trypanosomatid drug discovery. Curr Med Chem. 2014;21(15):1679–706. doi: 10.2174/09298673113209990139 23834165

