# Supplementary material for: Glycosomal ABC transporter 3 (GAT3) deletion enhances the oxidative stress responses and reduces the infectivity of Trypanosoma cruzi
Source: PLoS Negl Trop Dis. 2025 Sep 11;19(9):e0013479. doi: 10.1371/journal.pntd.0013479 (PMC12425183; doi:10.1371/journal.pntd.0013479)
Supplement: S1 Table — (DOCX) [file pntd.0013479.s002.docx]

**S1 Table**. List of primers used in this study.

| **PRIMER NAME** | **PRIMER SEQUENCE 5 ➛ 3** |
| --- | --- |
| Sg Scaffold Rv | AAAAGCACCGACTCGGTGCCACTTTTTCAAGTTGATAACGGACTAGCCTTATTTTAACTTGCTATTTCTAGCTCTAAAAC |
| TcDm28cGAT3_F1_up | GTGATGGAGGTCTTGTTGTCAGCGGAACACgtataatgcagacctgctgc |
| TcDm28cGAT3_R5_down | AACACGCGCCATAGTAATACGTTGTTTTTGccaatttgagagacctgtgc |
| sg3’_TcDm28c_GAT3 | gaaattaatacgactcactataggGATTGGGATTCTCTCAGCGGgttttagagctagaaatagc |
| sg5’_TcDm28c_GAT3 | gaaattaatacgactcactataggGGCGTTTCTGATTTGTCTCGgttttagagctagaaatagc |
| 5’ UTR Fw (P1) | GAGAAGAGGGTGCCGAATG |
| 3’ UTR Rv (P2) | GTCATACATTTGGGCACCAC |
| BLAST Rv (P3) | TTAGCCCTCCCACACATAACC |
| BLAST Fw (P4) | CAAGCCTTTGTCTCAAGAAGAATCCA |
| GAT3 Fw (P5) | GGCAACTTGGCAAACTGAAG |
| GAT3 Rv (P6) | CCCAATCCTGGTCACTCTTTATC |
| PURO Rv (P7) | TTACGTGGTGCAGACGTTTC |
| PURO Fw (P8) | GCGAGTCACAGAACTTCAAGAG |
| qPCR GAT1 Fw | CGTGAAACCAGGGATGAATCT |
| qPCR GAT1 Rv | TGATACGGCCACTCTGAATTG |
| qPCR GAT2 Fw | AGCGTGAGGGAGGATTAGAT |
| qPCR GAT2 Rv | ACACATGGCACGGATGATAG |
| qPCR GAT3 Fw | CGAAGGAGCTTGACTCGTTTA |
| qPCR GAT3 Rv | GCACCAACATACGGACTATCA |
| qPCR DNA Pol I Fw | AAACTGTTGCCGACCATGTG |
| qPCR DNA Pol I Rv | TTTCATACGAAGCGCAACCG |
