# Supplementary material for: Glycosomal ABC transporter 3 (GAT3) deletion enhances the oxidative stress responses and reduces the infectivity of Trypanosoma cruzi
Source: PLoS Negl Trop Dis. 2025 Sep 11;19(9):e0013479. doi: 10.1371/journal.pntd.0013479 (PMC12425183; doi:10.1371/journal.pntd.0013479)
Supplement: S2 Table — (DOCX) [file pntd.0013479.s003.docx]

**Supporting information**

**S2 Table.** *In vitro* trypanocidal activity, cytotoxicity, and selectivity index of selected compounds that interact with GAT3 against Tulahuen *T. cruzi* strain

| **Compound** | **EC_50_ (μM) ^1^** | **CC_50_ (μM) ^2^** | **Selectivity index (SI)^3^** |
| --- | --- | --- | --- |
| α-Tocopherol | inactive | - | - |
| Bumetanide | inactive | - | - |
| Glimepiride | 31.9 ± 7.99 | 196.30 | 6.16 |
| Benznidazole | 3.81 | 2381 | 625 |

^1^ Value of the effective concentration necessary to reduce growth by 50% (EC_50_) of the amastigotes and trypomastigotes of *T. cruzi*; ^2^Compound concentration that inhibits 50% of the L929 cell viability (CC_50_); ^3^CC_50_ L929/EC_50_ *T. cruzi*. EC_50_ and CC_50_ values were calculated by linear interpolation.
