# Supplementary figures and images for: Glycosomal ABC transporter 3 (GAT3) deletion enhances the oxidative stress responses and reduces the infectivity of Trypanosoma cruzi
Source: PLoS Negl Trop Dis. 2025 Sep 11;19(9):e0013479. doi: 10.1371/journal.pntd.0013479 (PMC12425183; doi:10.1371/journal.pntd.0013479)

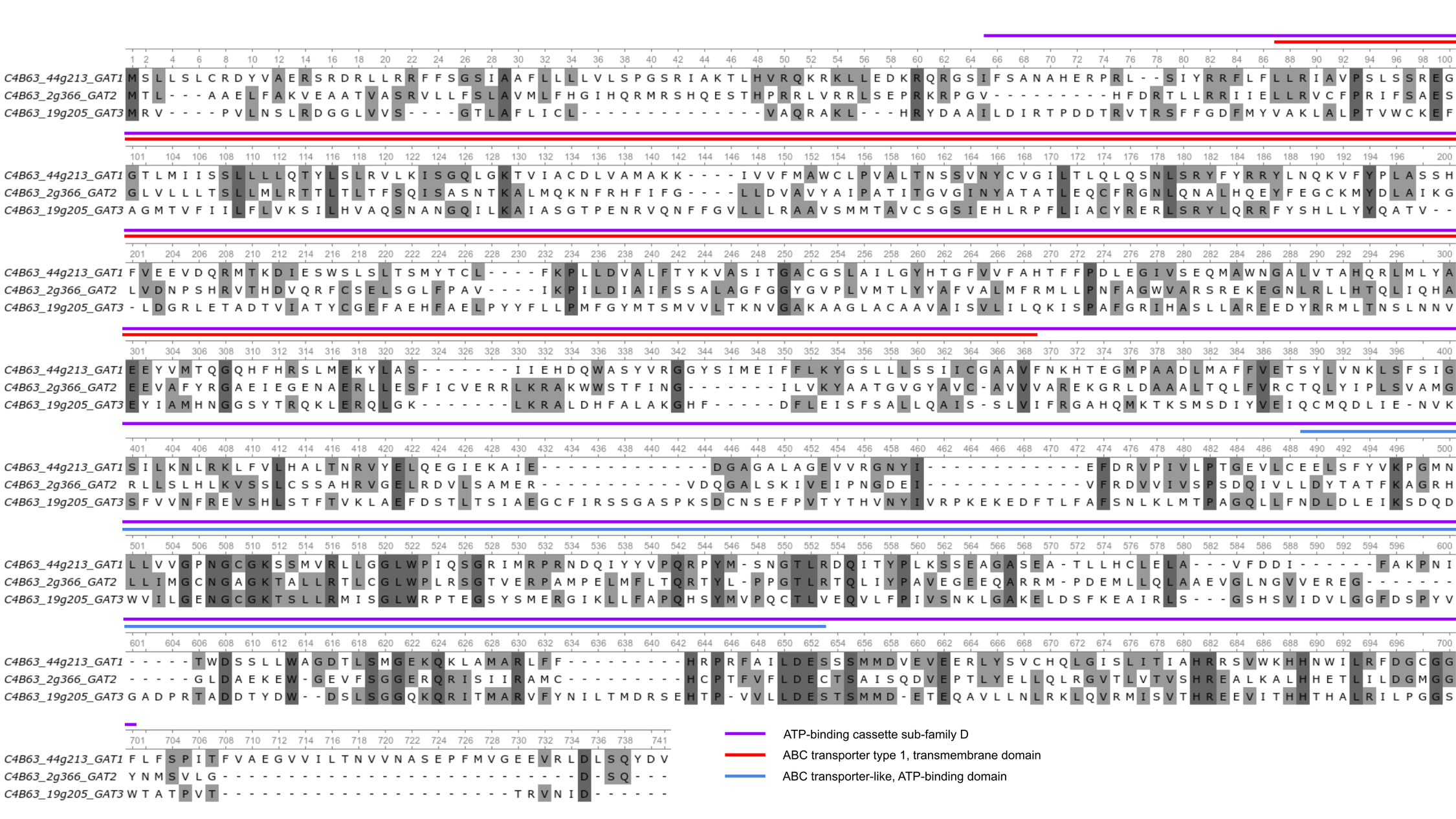

Supplement: S1 Fig — The amino acid sequences of TcGAT1 (C4B63_44g213), TcGAT2 (C4B63_2g366), and TcGAT3 (C4B63_19g205) from T. cruzi DM28c strain were aligned using MAFFT, highlighting conserved and variable residues. Conserved residues are shaded in dark gray, while less conserved positions are shaded in lighter gray. The domains were mapped with TcGAT1 as reference onto the alignment: ATP-binding cassette sub-family D domain (purple), ABC transporter type 1 transmembrane domain (red) and ABC transporter-like ATP-binding domain (blue). Gaps introduced during the alignment process are represented by dashes (-). The alignment illustrates the overall sequence conservation among the three transporters and highlights specific variations that may contribute to functional divergence. (TIF) [file pntd.0013479.s004.tif]

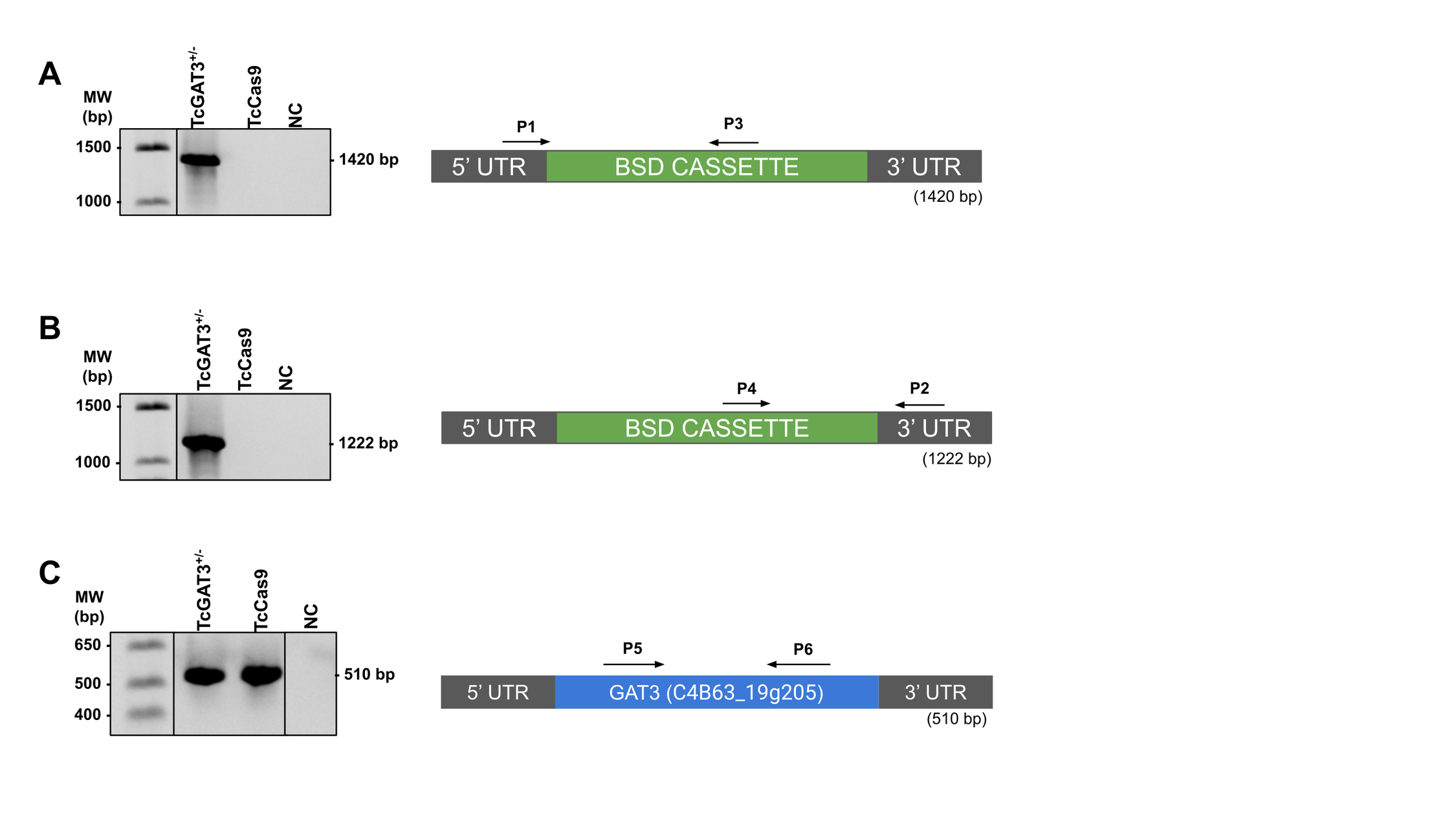

Supplement: S2 Fig — Correct integration of the resistance markers, (A and B) blasticidin S deaminase (BSD; 1420 bp and 1222 bp) was evaluated via PCR by annealing a primer in the 3′UTR and 5′UTR region adjacent to the cassette (primer P1 or P2) and another within each resistance marker sequence (primers P3 or P4 for BSD). (C) The fragment GAT3-coding sequence (510 bp) was amplified via PCR with the P5 and P6 primers. MW: molecular weight; NC: negative control; bp: base pair. (TIF) [file pntd.0013479.s005.tif]

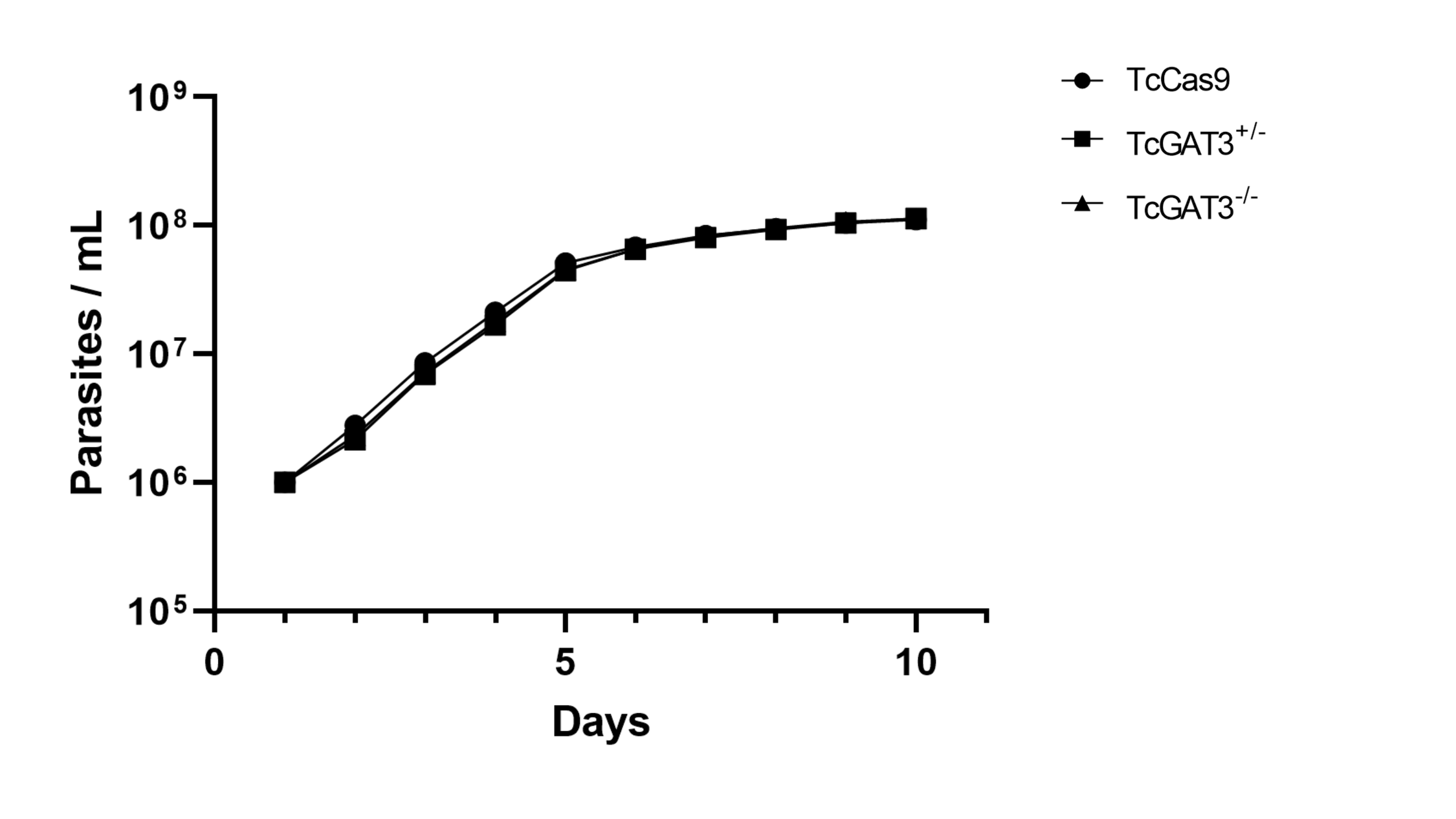

Supplement: S3 Fig — An initial inoculum of 2 x 10^6^ parasites per mL was prepared for the Cas9 parasites and mutant lines, which were counted every 24 h using the Z1 Coulter Counter, during 10 days. (TIF) [file pntd.0013479.s006.tif]
